# Fecal microbiota transplantation in patients with post-infectious irritable bowel syndrome: A randomized, clinical trial

**DOI:** 10.3389/fmed.2022.994911

**Published:** 2022-10-20

**Authors:** Sergii Tkach, Andrii Dorofeyev, Iurii Kuzenko, Oksana Sulaieva, Tetyana Falalyeyeva, Nazarii Kobyliak

**Affiliations:** ^1^Ukrainian Research and Practical Centre of Endocrine Surgery, Transplantation of Endocrine Organs and Tissues of the Ministry of Health of Ukraine, Kyiv, Ukraine; ^2^Shupyk National Medical Academy of Postgraduate Education, Kyiv, Ukraine; ^3^Medical Laboratory CSD, Kyiv, Ukraine; ^4^Educational-Scientific Center, “Institute of Biology and Medicine”, Taras Shevchenko National University of Kyiv, Kyiv, Ukraine; ^5^Endocrinology Department, Bogomolets National Medical University, Kyiv, Ukraine

**Keywords:** fecal microbiota transplantation, gut microbiota, dysbiosis, diarrhea, post-infectious irritable bowel syndrome

## Abstract

**Introduction:**

Research in recent years has shown the potential benefits of fecal microbiota transplantation (FMT) for irritable bowel syndrome (IBS). Acute infectious gastroenteritis is a well-established risk factor for developing such forms of IBS as post-infectious IBS (PI-IBS). However, the effective use of FMT in patients with IP-IBS has not yet been clarified.

**Aim:**

The study aimed to conduct a single-center, randomized clinical trial (RCT) to assess FMT’s safety, clinical and microbiological efficacy in patients with PI-IBS.

**Materials and methods:**

Patients with PI-IBS were randomized into two groups: I (standard-care, *n* = 29) were prescribed basic therapy, namely a low FODMAP diet, as well as Otilonium Bromide (1 tablet TID) and a multi-strain probiotic (1 capsule BID) for 1 month; II (FMT group, *n* = 30), each patient with PI-IBS underwent a single FMT procedure with fresh material by colonoscopy. All patients underwent bacteriological examination of feces for quantitative and qualitative microbiota composition changes. The clinical efficacy of treatment was evaluated according to the dynamics of abdominal symptoms, measured using the IBS-SSS scale, fatigue reduction (FAS scale), and a change in the quality of life (IBS-QoL scale).

**Results:**

FMT was associated with rapid onset of the effect, manifested in a significant difference between IBS-SSS points after 2 weeks of intervention (*p* < 0.001). In other time points (after 4 and 12 weeks) IBS-SSS did not differ significantly across both groups. Only after 3 months of treatment did their QoL exceed its initial level, as well value for 2 and 4 weeks, to a significant extent. The change in the ratio of the main microbial phenotypes in the form of an increase in the relative abundance of *Firmicutes* and *Bacteroidetes* was recorded in all patients after 4 weeks. It should be noted that these changes were significant but eventually normalized only in the group of PI-IBS patients who underwent FMT. No serious adverse reactions were noted.

**Conclusion:**

This comparative study of the results of FMT use in patients with PI-IBS demonstrated its effectiveness compared to traditional pharmacotherapy, as well as a high degree of safety and good tolerability.

## Introduction

Irritable bowel syndrome (IBS) is one of the most common gastroenterological diseases and affects 7–15% of the total population in developed countries (1). Although the reasons for the development of IBS are not fully understood. It is believed that its pathophysiology is based on a combination of psychopathological factors and intestinal dysfunction (2, 3). Nevertheless, more and more studies indicate that disorders of the gut microbiota play an important role in the pathogenesis of IBS by contributing to low-intensity inflammation of the intestinal mucosa and epithelial dysfunction, as do environmental factors such as prior intestinal infections (4–6).

Acute infectious gastroenteritis is a well-established risk factor for developing such forms of IBS as post-infectious IBS (PI-IBS) (7). A recent meta-analysis combined data from studies that confirmed that PI-IBS is a widespread pathology that develops in 10–45% of patients after acute intestinal infections (8, 9). On average, PI-IBS accounts for about 10% of all IBS cases, and more frequently develops after instances of bacterial gastroenteritis (as opposed to viral gastroenteritis).

Although there is no generally accepted definition of PI-IBS, it is believed that this form is characterized by the first onset of IBS symptoms (according to Rome IV criteria) after acute gastroenteritis in patients who did not have IBS before infection. The criteria for PI-IBS proposed by the Rome Foundation Working Team in 2019 (10). The division of PI-IBS into subtypes is based on stool consistency (according to the Bristol scale), with PI-IBS with diarrhea (PI-IBS-D) being the most common (11).

There are no specific treatment recommendations for PI-IBS; therefore, symptomatic treatment is usually carried out depending on the IBS subtype (12). Non-pharmacological methods include limiting or excluding foods rich in FODMAPs, which is especially useful in frequent diarrhea, common with PI-IBS (13, 14). Among extant medications, antidiarrheal agents, serotonin antagonists, antispasmodics, gut microbiota modulators, anti-inflammatory drugs, mast cell stabilizers, bile acid sequestrants, psychotropic drugs, and new opioid agonists can be utilized (15, 16). Some medicines have long been used in clinical practice, while others are still undergoing clinical trials.

Considering that PI-IBS is brought on by infection and gut microbiota may be associated with the onset of symptoms, the modification of altered gut microbiota with non-absorbable antibiotics such as rifaximin-α or probiotics is often employed as first-stage treatment (17, 18). Research in recent years has also shown the potential benefits of fecal microbiota transplantation (FMT) for IBS, which is the replacement of a sick recipient’s gut microbiota with fecal material from a healthy donor (19, 20). Even though the only officially approved indication for FMT at this time is recurrent *Clostridium difficile* infection, the effectiveness of FMT is nevertheless being studied for treating other gastrointestinal and non-gastrointestinal pathologies, including IBS (19, 21, 22). To date, several controlled and uncontrolled studies have been conducted to study the effectiveness of FMT for IBS, and most of them have demonstrated positive results (20, 23–26). However, the effective use of FMT in patients with PI-IBS has not yet been clearly clarified. The double-blind, randomized, placebo-controlled crossover trial shows that FMT is safe and feasible for patients with IBS but the treatment is not better than the placebo and it might even be worse (26). New randomized clinical trials (RCTs) are necessary to improve understanding of the therapeutic role of FMT in patients with IBS and IBS subgroups. So, the current study aimed to conduct a single-center, randomized clinical trial (RCT) to assess FMT’s safety, clinical and microbiological efficacy in patients with PI-IBS.

## Materials and methods

### Study design

This open-label, single-center, randomized clinical study was conducted to examine the effectiveness of FMT in patients with PI-IBS. The study protocol was in compliance with principles of the Declaration of Helsinki 1975 and approved by the Ethics Committee at Ukrainian Research and Practical Centre of Endocrine Surgery, Transplantation of Endocrine Organs and Tissues of the Ministry of Health of Ukraine (protocol number: 25/2020) and was registered in the Clinicaltrials.gov database under entry number NCT05461833. Before RCT initiated, its purpose and methods were clearly discussed with participants and after that all the patients voluntarily signed the informed consent.

All patients with PI-IBS were randomized into 2 groups in a 1:1 ratio. Randomization was carried out by an expert in statistics with blocks of four using a computer-generated list at www.randomization.com. The groups were homogeneous in terms of age, gender, and diagnosis. The patients of group I (standard-care, *n* = 29) were prescribed with basic therapy, namely a low FODMAP diet, as well as Otilonium Bromide (1 tablet TID) and a multi-strain probiotic (1 capsule BID) for 1 month. The “Probeez” probiotic (Organosyn Life Sciences Ltd., Kyiv, Ukraine) with total amount not less than 10.0 × 10^9^ CFU was used (*L. acidophilus* 2.0 × 10^9^ CFU, *L. rhamnosus* 1.5 × 10^9^ CFU, *L. plantarum* 1.5 × 10^9^ CFU, *L. reuteri* 1.0 × 10^9^ CFU, *L. casei* 1.0 × 10^9^ CFU, *B. bifidum* 1.0 × 10^9^ CFU, *Saccharomyces boulardii* 2.0 × 10^9^ CFU). In the second group (FMT group, *n* = 30), each patient with PI-IBS underwent a single FMT procedure with fresh material. The adherence to diet was assessed by low FODMAP food diary. The compliance with drugs and probiotic were assessed by returned amount of pills and capsules. Non-compliance was stated when participants received less than 85% of prescribed intervention.

### Participants selection

Participants were eligible for inclusion in the trial if they had an established diagnosis of the PI-IBS diagnosis in accordance with the Rome IV criteria (10). The severity of IBS was assessed by the IBS Symptom Severity Scale (IBS-SSS), in which mild, moderate, and severe IBS were defined in the ranges of 75–175, 175–300, and above 300 points, respectively. Other inclusion criteria were as follows: adult patients (age: 18–65 years) with moderate-to-severe disease activity (≥ 175 points on the IBS-SSS), normal appearing colon on colonoscopy with biopsy that did not reveal pathology and a signed informed consent form. Patients were excluded if they had a systemic disease, immunodeficiency, or previous treatment with immunomodulators; if pregnant or breastfeeding; with previous surgery on the abdominal cavity, with the exception of appendectomy, cholecystectomy, cesarean section and hysterectomy; with severe current disease (hepatic, renal, respiratory, or cardiovascular); with probiotic or antibiotic use within 8 weeks prior to study initiation; or any condition or circumstance that would, in the opinion of the investigator, prevent completion of the study or interfere with analysis of study results.

### Procedure

For our FMT procedures, we used fecal material from one super donor tested in accordance with the European Consensus on FMT that was published in the form of clinical guidelines for physicians in 2017 (27). A healthy 38-year-old Caucasian male was recruited as a super donor, inasmuch as he had no harmful habits, adhered to a healthy lifestyle, and had a BMI of 24.5 kg/m^2^. His fecal material has already been utilized in other FMTs that have proven effective in the treatment of recurrent *C. difficile* infection. The super donor underwent a physical examination, as well as studies and blood tests to exclude pathology of the gastrointestinal tract, metabolic or neurological disorders (complete blood count, blood glucose, electrolytes and inflammatory markers), liver tests and thyroid function tests, as well as serological screening tests for HIV, syphilis, and viral hepatitis A, B, and C. The results of his stool culture for the presence of pathogenic bacteria (*Shigella* spp., *Salmonella* spp., *Campylobacter* spp., *Yersinia* spp., and toxin-producing *Clostridioides difficile*), rotaviruses, helminth eggs, and parasites were also negative. The superdonor had no blood transfusions, accidental injuries, parasitosis, zoonotic infections and infectious diseases, travel to tropical countries and countries with a high risk of developing infectious diseases or traveler’s diarrhea, vaccination with live (attenuated) virus. In the previous 6 months, he has not received treatment with antibiotics, immunosuppressants, chemotherapy, proton pump inhibitors. His stool culture indicated the absence of gut dysbiosis. Super donor fecal samples were tested every 2 months and remained normobiotic with minor variations in the quantitative composition of gut bacteria.

FMT were prepared as follows: 50–80 g of freshly delivered feces were mixed with 200 mL of isotonic saline and 50 mL of 85% glycerol, homogenized in a blender for 60 s, filtered through a 0.5 mm mesh steel strainer, drawn on 50 mL sterile Luerlock syringes, and sealed.

An appropriately prepared fresh stool suspension from a healthy donor was administered to all patients a single time during a colonoscopy (through a probe inserted into the working channel of the endoscope) while patients were under the effects of intravenous anesthesia.

### Outcomes assessment

All patients underwent a comprehensive laboratory and instrumental examination, including general clinical and biochemical blood tests (liver function tests, thyroid hormones, serological examination for celiac disease, electrolytes), fecal examination for calprotectin, helminth eggs and parasites, abdominal ultrasonography, gastroduodenoscopy and colonoscopy with segmental biopsy (to exclude inflammatory bowel diseases).

The primary outcome was the difference in disease severity between the FMT and the standard care group at 12 weeks, as measured by IBS-SSS. Also the response rate was assessed which defined as decrease of ≥ 50 points on the IBS-SSS. The IBS-SSS evaluates retrospectively the intensity of IBS symptoms during the past 10 days: abdominal pain, distension, stool frequency and consistency, and interference with life in general. Each item is scored on a visual analogue scale from 0 to 100 and the score for all five summed. A total score of 175–300 is deemed moderate severity and a score of more than 300 is deemed severe (28).

Secondary outcomes were the reduction of fatigue (according to fatigue assessment scale (FAS) questionnaire), changes to patients’ quality of life (according to IBS Quality of Life Scale (IBS-QoL) questionnaire) after 12 weeks of treatment. The IBS-QoL is a 34-item measure assessing the degree to which IBS interferes with a patient’s QOL. Each item is rated on a 5-point Likert scale, all items are summed, and the total score is converted to a 100-point scale, with high scores indicating a better QoL (28, 29). The FAS questionnaire comprises 10 questions with 5-point-scale answers varying from never to always. Five of these questions measured physical fatigue and the other five measured mental fatigue (30). Study questionnaires were administered by members of the study team trained in administering and overseeing completion of the questionnaires. Participants completed study questionnaires at baseline and 2, 4, and 24 weeks after randomization.

All patients underwent bacteriological examination of feces for quantitative and qualitative microbiota composition by Epstein-Litvak R.V. and Vilshanskaya F.L. in term of secondary outcome. The percentage of patients in each group characterized by a decrease below the normal content of symbiotic bacteria *Bifidobacterium* (less than 10^7^ CFU/g), *Lactobacillus* (less than 10^7^ CFU/g), *Escherichia coli* (*E. coli*) with normal properties (less than 10^6^ CFU/g) and increase in the content of *E. coli* with altered properties (more than 10^6^ CFU/g), pathogenic enterobacteria (not normally detected) and *Candida* (more than 10^4^ CFU/d) was determined (18). Given that some patients were characterized by changes in one component of the microflora and others were within normal limits, we also determined the percentage of patients characterized by changes in the content of at least one of the representatives of microbiocenosis.

The gut microbiota of all patients was studied before and 1 month after treatment at the level of the main microbial phylotypes by determining the DNA *Firmicutes, Bacteroidetes* and *Actinobacteria* in stool samples using a quantitative real-time polymerase chain reaction (PCR) (qRT-PCR). For this, samples of fresh feces were placed in a special container by each patient. An aliquot of feces was taken within 10 min after defecation, immediately frozen, and stored at –20°C until DNA isolation using the phenol-chloroform method according to protocol. DNA was eluted in 200 μl of buffer, and the amount and quality of DNA was measured using a NanoDrop ND-8000 (Thermo Scientific, USA). Samples with a DNA concentration of fewer than 20 ng or with a 260:280 fluorescence ratio of less than 1.8 were either subjected to ethanol precipitation to become concentrated or further purified according to quality standards. Various taxa were quantified by qPCR using primers targeting the 16S rRNA gene specific for *Firmicutes, Actinobacteria* and *Bacteroidetes* as well as universal primers. The primer sequences presented at Table 1. PCR reaction was performed in real-time thermal cycler Rotor-Gene 6000 (QIAGEN, Germany). The PCR reaction conditions consisted of an initial denaturing step of 5 min at 95°C, 30 cycles of 95°C for 15 s, annealing for 15 s and 72°C for 30 s, and a final elongation step at 72°C for 5 min. Every PCR reaction contained 0.05 units/μl of Taq polymerase (Sigma Aldrich), 0.2 mM of each dNTP, 0.4 μM of each primer, 1 × buffer, ∼10 ng of DNA and water to 25 μl (31).

**TABLE 1:** Primers used for PCR.

	Primers
*Bacteroidetes*	798cfbF AAACTCAAAKGAATTGACGG (forward) cfb967R GGTAAGGTTCCTCGCGCTAT (reverse)
*Firmicutes*	928F–firm TGAAACTYAAGGAATTGACG (forward) 1040FirmR ACCATGCACCACCTGTC (reverse)
*Actinobacteria*	Act920F3 TACGGCCGCAAGGCTA (forward) Act1200R TCRTCCCCACCTTCCTCCG (reverse)
Universal bacterial 16S rRNA sequences	926F AAACTCAAAKGAATTGACGG (forward) 1062R CTCACRRCACGAGCTGAC (reverse)

Adverse reactions due to FMT were assessed daily over a period of 3 days, and then weekly over a period of 4 weeks.

### Sample size calculation

The sample size was calculated using WINPEPI 11.65 (Brixton Health, Israel) software based on the previously published study (23). Estimating that response rates of 50% in the active group would be clinically relevant, we calculated that we would need 50 participants in a balanced two-group design (α = 0⋅05; 1-β = 0⋅80). To allow for dropouts, we initially planned to enroll 60 participants to include in intention-to-treat analysis.

### Statistical analysis

Statistical analysis was performed using the standard software SPSS version 20.0 (SPSS, Inc., Chicago, Illinois) and GraphPad Prism, version 6.0 (GraphPad Software, Inc., La Jolla, CA, USA). Analyses were done according to the intention-to-treat principle, excluding participants without data from the analyses of all clinical endpoints, who did not undergo treatment and participants diagnosed with any other disease at 12 weeks. Quantitative changes were presented as the mean and standard deviation (Ì ± SD), qualitative changes were presented as%. In order to prove the normal distribution hypothesis, Kolmogorov-Smirnov one-sample test was used. To estimate the difference of the incoming quantitative data χ2 criterion was used. A paired *t*-test and a repeated measure analysis of variance (RM-ANOVA), was used to determine, within each group, the difference between the initiation of therapy and 2, 4 weeks and end of the trial. The participants’ outcomes changes after the initiation of treatment and end of the trial were compared by paired sample *t*-tests. Analysis of covariance (ANCOVA) was used to identify any differences between the two groups after intervention, adjusting for baseline values. Differences between groups were considered significant at a value of *p* < 0.05.

## Results

**FIGURE 1:**
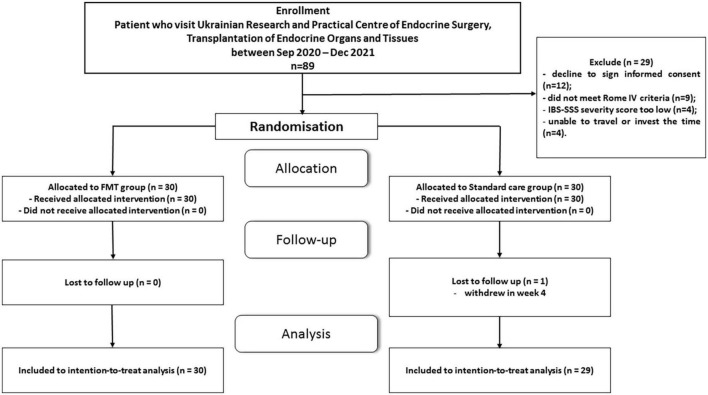
Consolidated standards of reporting trials (CONSORT) flow chart–trial protocol.

Recruitment started in September 2020 and continued until December 2021 at the Ukrainian Research and Practical Centre of Endocrine Surgery, Transplantation of Endocrine Organs and Tissues of the Ministry of Health of Ukraine. For the primary analysis, 89 patients were selected. After careful consideration for compliance with the inclusion/exclusion criteria, 13 patients were not eligible. The main reasons were IBS-SSS severity score to low (≥ 175 points) and did not meet Rome IV criteria (Figure 1). A face-to-face conversation was held with all other potential participants explaining the main study criteria, purpose and methodology. After consideration of the proposal, 12 patients refused to give their informed consent and 4 unable to travel or invest the time. At the end of the enrolment period, with possible bias adjustment, 60 patients with PI-IBS were chosen to be included in the study. All patients were equally distributed in a random order to FMT or standard care group. One randomly assigned participant in the standard care group withdrew his informed consent at week 4. This left 59 participants for the final modified intention-to-treat analysis. A CONSORT flow chart with a general protocol schedule is shown in Figure 1.

There was no statistically significant difference between the groups in baseline clinical and demographic characteristics of enrolled patients (Table 2). A total of 59 patients (38 women, 21 men) with PI-IBS with diarrhea who ranged from 22 to 58 years old (mean age—41.3 ± 12 years) were examined.

**TABLE 2:** Baseline clinical parameters in examined patients (M ± SD or %).

Baseline characteristics	Standard care group (*n* = 29)	FMT group (*n* = 30)	*p*
**Gender (male/female)**	11/18	10/20	0.310
**Age, years**	40.1 ± 12.1	42.4 ± 11.4	0.360
**IBS duration, years**	3.3 ± 0.9	4.1 ± 1.5	0.110
**Smoking status, *n* (%)**	10 (34.5%)	12 (40.0%)	0.114
**Body mass index (BMI), kg/m^2^**	24.9 ± 2.8	25.5 ± 2.9	0.545
**IBS-SSS, points**	309.37 ± 56.73	306.56 ± 55.72	0.848
**FAS, points**	31.65 ± 5.52	31.3 ± 4.45	0.786
**IBS-QoL, points**	117.03 ± 15.75	117.46 ± 16.09	0.917
**Dysbiosis, *n* (%)**	25 (86.2%)	26 (86.6%)	0.926

**FIGURE 2:**
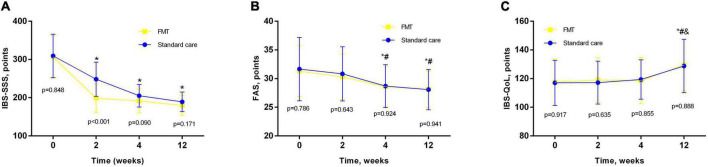
Main outcomes analysis in different timepoints. **(A)** IBS-SSS; **(B)** FAS scale; **(C)** IBS-QoL scale. Data expressed as mean ± SD. RM-ANOVA was used to identify any differences within groups. *p* indicates the difference between groups at the same timepoint. *, as compared to baseline; #, compared to 2 week; &, compared to 4 week.

The clinical efficacy of the treatment across both groups of patients is shown in Figure 2. The study showed that treatment was effective in most patients in both groups of patients with PI-IBS. The systemic severity of IBS symptoms in both groups of patients progressively decreased, exhibiting a significant decrease after just 2 weeks compared to baseline values (*p* < 0.01) and reaching a maximum after 12 weeks from the start of treatment. The differences in IBS-SSS scores between baseline and all intermediate time points within each group were all statistically significant (*p* < 0.001 for FMT; *p* < 0.001 for standard care). FMT was associated with rapid onset of the effect, manifested in a significant difference between IBS-SSS points after 2 weeks of intervention (*p* < 0.001). In other time points (after 4 and 12 weeks) IBS-SSS did not differ significantly across both groups (Figure 2A). The number of responders in both groups of patients was significantly higher when assessed 12 weeks after the start of treatment as compared to the assessment carried out 2 weeks after the start of treatment. The clinical response rate was similar between groups. Moreover, 19 (65.5%) of 29 participants receiving standard care treatment vs. 20 (66.7%) of 30 receiving the FMT showed a response of a decrease in IBS-SSS of more than 50 points at 3 months after FMT (*p* = 0.572). In between group analysis IBS-SSS did not changed significantly (*p* = 0.705; Table 3).

**TABLE 3:** Outcomes compared within and between groups.

	Standard care group (*n* = 29)	FMT group (*n* = 30)
**IBS-SSS**		
Baseline value	309.37 ± 56.73	306.56 ± 55.72
Week 12 value	189.17 ± 25.80	179.80 ± 26.1
*P*-value for change from baseline	<0.001	<0.001
Between-group *p*-value 0.705		
**FAS**		
Baseline value	31.65 ± 5.52	31.3 ± 4.45
Week 12 value	28.06 ± 3.51	28.13 ± 3.15
*P*-value for change from baseline	<0.001	<0.001
Between-group *p*-value 0.515		
**IBS-QoL**		
Baseline value	117.03 ± 15.75	117.46 ± 16.09
Week 12 value	128.79 ± 18.63	129.46 ± 17.88
*P*-value for change from baseline	<0.001	<0.001
Between-group *p*-value 0.948		

For within group analysis paired sample t-tests was used. ANCOVA was used to identify any differences between the two groups after intervention, adjusting for baseline value.

Beginning at 4 weeks of treatment, the severity of fatigue (both physical and mental) according to the FAS scale also decreased significantly (*p* < 0.001) as compared to the baseline of both groups of patients (Figure 2B). There was no significant improvement in the quality of life in terms of IBS-QoL 2 and 4 weeks after the start of treatment in both groups of patients. Only after 3 months of treatment did their QoL exceed its initial level, as well value for 2 and 4 weeks, to a significant extent (Figure 2C). In between group analysis both scales did not changed significantly (*p* = 0.515 for FAS; *p* = 0.948 for IBS-QoL; Table 3).

Changes in the qualitative and quantitative composition of the gut microflora were recorded in most patients with PI-IBS before the start of treatment and contingent upon the severity of the patient’s IBS. A qualitative analysis of the gut microbiota showed a higher frequency of seeding of various *Staphylococci* and *Streptococci* with pathogenic properties (*S. Aureus* and *S. Epidermidis* (heme +) and also *E. coli* (heme +), lactose-negative *E.coli*, *Klebsiella* spp., *Proteus* spp., and *Enterobacte*r spp. from the feces of patients with PI-IBS. Furthermore, a significant decrease in the quantitative level of *Bifidobacterium* spp. (81.3% of patients) and *Lactobacillus* spp. (71.9% of patients) was recorded. One month after the start of treatment, both groups of patients showed a significant decrease in both the frequency of intestinal dysbiosis as well as the degree of its severity as compared to baseline values (*p* < 0.05), which was significantly more frequent in the group of PI-IBS patients that received FMT (Table 4).

**TABLE 4:** Frequency and severity of intestinal dysbiosis in patients before and 4 weeks after treatment.

Gut dysbiosis severity	Standard care group (*n* = 29)		FMT group (*n* = 30)	
	Baseline	After 4 weeks	Baseline	After 4 weeks
**1st degree, *n* (%)**	3 (10.3)	10 (34.5)*	4 (13.3)	8 (26.7)**
**2nd degree, *n* (%)**	17 (58.6)	2 (6.9)*	14 (46.7)	2 (6.7)*
**3rd degree, *n* (%)**	5 (17.3)	2 (6.9)**	8 (26.7)	–
**Total, *n* (%)**	25 (86.2%)	14 (48.3)*	26 (86.7%)	10 (33.4)**

*p < 0.05 as compared to the baseline; **p < 0.05 between groups.

Patients with PI-IBS showed an increase in other opportunistic flora, mainly *Proteobacteria*. Four weeks after the start of treatment, a change in the ratio of the main microbial phenotypes was recorded in the form of an increase in the relative abundance of *Firmicutes* and *Bacteroidetes* in both groups of patients. It should be noted that these changes were significant but eventually normalized only in the group of PI-IBS patients who underwent FMT. It should also be noted that the levels of *Actinobacteria* and other conditionally pathogenic flora in patients of this group also corresponded to norms after therapy. In contrast, tendencies toward increased *Firmicutes* and *Bacteroidetes* and decreased *Actinobacteria* were noted in the patients of the standard care group (Table 5). The high microbiological efficiency of FMT in patients with PI-IBS may be associated with modified metabolic activity of the gut microbiota due to the high content of regulatory molecules and metabolites in the super donor’s feces, which led to a significant increase in the level of *Firmicutes* and *Bacteroidetes* as well as increased the synthesis of short-chain fatty acids, in particular butyrate.

**TABLE 5:** Contents of the main phylotypes of microorganisms in patients with PI-IBS at baseline and 4 weeks after treatment (%).

Microbial phylotype (%)	Standard care group (*n* = 29)		FMT group (*n* = 30)	
	Baseline	After 1 month	Baseline	After 1 month
***Firmicutes***	27 (23–32)	30 (26–35)	26 (23–31)	36 (28–44)*
***Bacteroidetes***	36 (23–44)	40 (33–46)	36 (32–40)	46 (32–58)*
***F/B* ratio**	0.75 (0.5–1.0)	0.75 (0.5–1.0)	0.7 (0.5–0.9)	0.78 (0.7–0.9)
***Actinobacteria***	26 (22–34)	20 (4–8)	26 (21–35)	14 (8–19)*
**Other**	11 (8–14)	10 (7–14)	12 (9–15)	4 (32–58)*

Medians and interquartile ranges are presented; *p < 0.05 as compared to baseline.

Adverse events (AE) likely related to FMT were stated in patients with PI-IBS. No serious adverse reactions were noted. Mild adverse reactions were observed in 8 patients (26.6%). Most often, there was a short-term increase in abdominal pain and bloating (6 patients, 20.0%), as well as diarrhea (5 patients, 16.6%). Less often, patients exhibited constipation (3 patients, 10.0%) and nausea (2 patients, 6.7%).

Thus, our comparative study of the results of FMT use in patients with PI-IBS demonstrated its effectiveness compared to traditional pharmacotherapy, as well as a high degree of safety and good tolerability.

## Discussion

Recent studies have shown that PI-IBS is a fairly common pathology in about 1 in 10 individuals with acute infectious gastroenteritis (8, 10). Since acute infectious gastroenteritis is one of the main risk factors for IBS, and the latter can be objectively identified after infection, this allows us to refute the claims that IBS is a cryptogenic condition. The main risk factors for developing PI-IBS include female, a young age, as well as certain psychological factors before or during acute infectious gastroenteritis (anxiety, depression, somatization, etc.). The severity and duration of infectious gastroenteritis must also be considered. The natural course of PI-IBS suggests that its manifestations subside over time and the overall prognosis may be better than with IBS as a whole (9). The pathophysiology of PI-IBS is multifactorial and includes motility disorders, visceral hypersensitivity, intestinal dysbiosis, immune activation, changes in enteroendocrine cells, and genetic factors. Insofar as specific therapeutic recommendations for PI-IBS have not yet been developed, its treatment is similar to the treatment of various subtypes of IBS (10).

In most patients with IBS, gut microbiota diversity is reduced, an increase of *Enterobacteria* is exhibited alongside a comparatively reduced quantity of *Bifidobacteria* and *Lactobacilli*, and the ratios of the main gut microbiota phylotypes are upset (28). Since gut dysbiosis is currently considered an important pathogenetic factor of IBS, various therapeutic strategies associated with gut microbiota modification (including FMT) have recently been the subject of intensive study (19). There are several prospective and RCTs of FMT’s effectiveness in IBS. For example, ninety patients with diarrhea IBS (IBS-D) or mixed IBS (IBS-M) were blindly randomized (2:1) into groups and received either FMT or a placebo. After 3 months, clinical response was detected in the FMT group (mixed material from 2 donors) in 65% of patients compared with 43% in the placebo group (23). Holvoet et al. examined 64 patients who had IBS without constipation (24). Patients were blindly randomized (2:1) to receive either FMT from 2 donors (*via* colonoscopy) or FMT from their own feces (placebo).

In the FMT-group was a considerable reduction of dyspeptic symptoms as compared to the placebo group. Microbiome analysis revealed that patients with positive response to FMT had both a higher baseline concentration of *Streptococcus* and enrichment of the entire gut microbiome than non-responders. Halkjær et al. studied the effectiveness of FMT in capsules for IBS vs. a placebo (25, 32). However, using capsules in patients improved symptoms and microbial diversity compared to control. Three American centers investigated the efficiency of capsules with FMT in patients with IBS-D in the double-blind, placebo-controlled RCT (26). Participants were randomized to receive 25 capsules with FTM (donor stool 0.38 g) per day for 3 days or placebo capsules in the appropriate amount. The efficacy of therapy was not proven after 12 weeks by the IBS severity index. So, the investigators concluded that further studies are needed to establish the effectiveness of FMT in patients with IBS. Although the authors assumed that PI-IBS patients might benefit most from FMT because these patients are likely to develop gastrointestinal symptoms directly from an altered gut microbial community after acute gastroenteritis (26). Indeed, in our study we confirmed this hypothesis. We conducted a corresponding study on FMT’s efficacy and safety in PI-IBS compared to traditional pharmacotherapy. By design, the study was a single-center, double-blind, randomized, and comparative study conducted in parallel groups of patients with PI-IBS. Limitations of the study included the absence of double-blind control, a relatively small number of patients with only one phenotype (IBS-D), and the scheduling of only a single FMT procedure with fresh material. Nevertheless, as a result of the study, we found that a single FMT treatment in patients with PI-IBS was effective for more than 60% of patients, and the procedure’s efficacy and safety were comparable to the results of traditional 4-week pharmacotherapy. Our data are consistent with the results of other studies (23–25). The effectiveness of FMT manifested clinically after just 2 weeks and increased gradually for up to 3 months of observation. One advantage of this method was its effectiveness on a microbiological level, which manifested in a decrease in the frequency and severity of intestinal dysbiosis, an increase in the gut microbiota diversity, and a tendency toward normalizing the its main phylotype ratios after just 4 weeks. Mild side effects of FMT occurred relatively infrequently and were both short-term and transient. Therefore, they did not have any significant clinical significance.

A significant factor in the effectiveness of FTM is the method and form of administration. The microbial diversity was higher in the FMT group (capsules), but the symptomatic improvement was better in the control group (25, 26). However, in single-blind randomized trials patients established a significant reduction of dyspeptic phenomena in the experimental group (receive FMT via colonoscopy from 2 donors) in comparison with the control group (FMT from their feces). Also, the improvement of gut microbiota was observed in the patients of the experimental group (24).

Notably, the results of the aforementioned studies were combined in a meta-analysis. So, in 4 (33), 5 RCT (34) and 7 (35, 36) meta-analyses established that FMT doesn’t improve overall IBS symptoms in comparison with placebo. Conversely, a different meta-analysis assessed data from single-arm trials (SATs) and RCTs separately. In SATs, was established statistically significant changes in the improvement of the condition of patients with IBS by 60% (95% CI 49.1–69.3). But in RCTs, the effectiveness of the use of FTM has not been proven (RR = 0.93 (95% CI 0.50–1.75) or changes in the IBS-SSS (37). However, FMT is a promising new strategy in the treatment of IBS, but delivery systems are lacking, as classic FMT shows promise while capsule delivery does not. The benefits of single-dose FMT using colonoscopy and nasojejunal tubes compared with placebo and a reduction in the likelihood of improvement of multiple-dose capsule were observed in sub-analysis (33, 34). In contrast to the previous meta-analysis, 2 recent ones published in 2022 similarly do not show significant global improvement in patients with IBS. Nonetheless, FMT operated by invasive routes *via* colonoscopy or gastroscope significantly improved global IBS symptoms (35, 36).

## Conclusion

This randomized comparative study on the efficacy and safety of FMT (fresh material) in patients with PI-IBS showed that even a single administration significantly affects the IM by reducing the frequency and severity of dysbiotic disorders. Furthermore, it is accompanied by significant clinical improvement in most patients that lasts for up to 3 months of observation and is comparable in effectiveness to pharmacotherapeutic methods. As subject to all provisions of the European Consensus on FMT, FMT is safe, well tolerated by patients, and can be administered repeatedly. Before FMT is officially recommended for the treatment of diseases other than *Clostridium difficile* infection, including IBS and PI-IBS, there are still many questions that must be answered regarding issues that can potentially affect the effectiveness of FMT. These questions relate to both the nature of this FMT-sensitive pathology and the initial state of the recipient patient’s IM, as well as the selection of suitable donors, the routes of material administration and the frequency of procedures. We sincerely hope that these questions will be answered in the very near future.

## Data availability statement

The raw data supporting the conclusions of this article will be made available by the authors, without undue reservation.

## Ethics statement

The studies involving human participants were reviewed and approved by the Ethics Committee at Ukrainian Research and Practical Centre of Endocrine Surgery, Transplantation of Endocrine Organs and Tissues of the Ministry of Health of Ukraine. The patients/participants provided their written informed consent to participate in this study.

## Author contributions

ST, AD, and NK contributed the conceptualization and the original idea of this manuscript. ST, AD, OS, IK, and NK contributed the methodology and reviewed the literature. ST, AD, and TF were involved in validation and revised and validated the literature findings. IK and OS performed the formal analysis. ST and AD conducted the investigation. ST and NK were involved in data curation, writing—original draft preparation, review and editing, and supervision. ST and IK performed the visualization and contributed to project administration. All authors contributed to the article and approved the submitted version.
